# SADiff: Coronary Artery Segmentation in CT Angiography Using Spatial Attention and Diffusion Model

**DOI:** 10.3390/jimaging11060192

**Published:** 2025-06-11

**Authors:** Ruoxuan Xu, Longhui Dai, Jianru Wang, Lei Zhang, Yuanquan Wang

**Affiliations:** School of Artificial Intelligence, Hebei University of Technology (HeBUT), Tianjin 300401, China; 18273033980@163.com (R.X.); 202332803032@stu.hebut.edu.cn (L.D.); 202122802005@stu.hebut.edu.cn (J.W.)

**Keywords:** coronary artery, image segmentation, diffusion model, multi-scale spatial attention

## Abstract

Coronary artery disease (CAD) is a highly prevalent cardiovascular disease and one of the leading causes of death worldwide. The accurate segmentation of coronary arteries from CT angiography (CTA) images is essential for the diagnosis and treatment of coronary artery disease. However, due to small vessel diameters, large morphological variations, low contrast, and motion artifacts, conventional segmentation methods, including classical image processing (such as region growing and level sets) and early deep learning models with limited receptive fields, are unsatisfactory. We propose SADiff, a hybrid framework that integrates a dilated attention network (DAN) for ROI extraction, a diffusion-based subnet for noise suppression in low-contrast regions, and a striped attention network (SAN) to refine tubular structures affected by morphological variations. Experiments on the public ImageCAS dataset show that it has a Dice score of 83.48% and a Hausdorff distance of 19.43 mm, which is 6.57% higher than U-Net3D in terms of Dice. The cross-dataset validation on the private ImageLaPP dataset verifies its generalizability with a Dice score of 79.42%. This comprehensive evaluation demonstrates that SADiff provides a more efficient and versatile method for coronary segmentation and shows great potential for improving the diagnosis and treatment of CAD.

## 1. Introduction

Cardiovascular disease stands as a primary global mortality factor [1], with a declining age of onset observed in recent years. The accurate extraction of coronary artery structures from images enables healthcare experts to determine the extent and location of coronary stenosis, facilitating more insightful intervention decisions. Moreover, rigorous coronary segmentation is essential for applying non-invasive computational FFR techniques [2]. However, the manual segmentation of coronary arteries is labor-intensive, costly, and requires significant medical expertise. Thus, there is an unmet clinical need for an efficient automated coronary segmentation tool to better identify and manage coronary artery disease.

Segmenting the coronary artery is inherently challenging due to the following: (1) the minute presence of coronary artery voxels in CTA images, accounting for a mere 0.16%, resulting in a pronounced imbalance between foreground and background; (2) the inherent variability of coronary artery tissues across individuals in terms of structure and morphology, demanding highly adaptive and generalized algorithms; and (3) the influence of external factors like motion artifacts, calcified plaque, and blood flow, which introduce noise and artifacts, further complicating the segmentation process.

Historically, coronary artery segmentation has evolved significantly with the advent of deep learning methods, which have demonstrated superior adaptability and efficiency compared to traditional approaches. Deep learning techniques, particularly convolutional neural networks (CNNs), have become the cornerstone of modern segmentation tasks due to their ability to automatically learn hierarchical features from large datasets. For instance, architectures like U-Net and its variants have been widely adopted for medical image segmentation, offering improved accuracy and robustness in handling complex anatomical structures such as coronary arteries [3,4]. Despite these advances, the challenges of the limitations of symmetric convolution in capturing tubular features, the memory constraints of larger kernels, and the impact of motion artifacts on segmentation accuracy remain. Recent innovations, such as attention mechanisms and transformer-based models, have further enhanced the performance of deep learning methods in this domain [5,6]. However, fundamental limitations persist in current approaches: conventional symmetric convolutional operations struggle to effectively model the elongated tubular structures of coronary arteries; the computational demands of processing high-dimensional medical volumes with large receptive fields remain prohibitive; and unavoidable motion artifacts in clinical imaging continue to degrade segmentation accuracy.

To overcome these challenges, we propose a novel segmentation model. Firstly, it addresses the category imbalance problem by pinpointing the ROI of coronary arteries and eliminating unnecessary noisy regions through the DAN. The DAN constructs spatial attention using dilated convolution to expand the convolution’s receptive field and uses the clDice loss function to preserve the topology of the coronary arteries. Subsequently, an accurate coronary segmentation mask is derived by fusing features from the DAN, SAN and generative subnet. The generation subnet maintains 3D consistency in generating 2D probabilistic masks through two mechanisms: first, the 3D ROI features provided by the DAN are utilized to guide the generation at the slice level to ensure anatomical soundness; and second, the inter-slice feature propagation of the vascular trajectories between slices is strengthened by the stripe convolution of the SAN and the topology preserving loss. Experiments show that this design effectively realizes the fusion of a 2D mask and 3D structure, ensuring the complete preservation of vascular branches in complex regions. A dual-encoder setup preserves local semantic information through a gated attention mechanism in the generative subnet. The SAN employs an encoder-feature fusion-decoder design, coupled with a new multi-scale strip spatial attention mechanism, to improve the accuracy of coronary segmentation. Moreover, a unique parallel architecture enhances the network’s representational capability by aggregating multi-sensory field feature maps produced by varied striped convolutional layers. In summary, the main contributions of this paper include the following:

1. We propose a novel cascade coronary segmentation model, amalgamating both generative and discriminative networks. The DAN extracts coronary ROI information and 3D information. The accuracy of extracted 2D information is improved by introducing diffusion model learning. The SAN refines the segmentation based on 3D and 2D information and finally obtains excellent results.
2. The SAN tackles the semantic gap in encoder–decoder frameworks through a tubular-structure-specific encoder-feature fusion-decoder architecture. To enhance the model’s ability to capture multi-scale information, we introduce a novel multi-stage gated attention mechanism within the SAN.
3. We propose two plug-and-play mechanisms for spatial attention. The first expands the receptive field through dilated convolution, allowing for the rapid retrieval of coronary voxel information. The second takes into account both global and local features through strip convolution, with a particular focus on capturing the tubular features of coronary arteries.
4. The dual dataset experiments with the public dataset ImageCAS (Dice = 83.48%) and the private dataset ImageLaPP (Dice = 79.42%) verified the advantages of the model in noise robustness and cross-center generalization, and its performance is significantly better than that of the mainstream methods, such as U-Net3D, TransU-Net, etc., which provides a reliable solution for the clinical automation segmentation.

The paper is structured as follows: Section 2 outlines the related literature. Section 3 explains the methodology of implementation in detail. Section 4 reports the experimental setup and results, followed by a discussion. Finally, Section 5 concludes the work.

## 2. Related Works

### 2.1. Cardiac Coronary Artery Segmentation

Deep learning stands as the prevailing method in addressing coronary segmentation challenges. Shen and Ye et al. [7] were among the first to employ a full convolutional network (FCN) for coronary segmentation in CCTA and incorporated an attention mechanism for enhanced segmentation precision. Pan et al. [8] honed the segmentation accuracy by evolving the FCN into U-Net. Harms et al. [9] capitalized on a target detection network, R-CNN [10], to swiftly pinpoint the rRegion of interest (ROI). Huang et al. [11] harmonized predictions with attention network outcomes, later applying a level set function to blend and amplify the results. Dong et al. [12] incorporated a dilated convolution to broaden the receptive field and utilized a multi-stage neural network to ensure the retention of vessel morphology and continuity. Notably, Zhang et al. [13] proposed a nested dense network with dilated convolutions for cardiac MR segmentation, which inspired the dilated attention in our DAN module. Sun et al. [14] amalgamated transformer and convolutional neural networks within a two-stage network to refine segmentation outcomes within the heart. Dong et al. [15] boosted the network’s feature capturing potential through the integration of various attention modules. ImageCAS [16] introduced a publicly accessible CCTA dataset and proffered a multi-scale patch fusion method. DSCNet [17] adeptly segmented intricate tubular structures like blood vessels via multi-view feature fusion and dynamic snake convolution techniques.

### 2.2. Diffusion Model

The DDPM diffusion model [18] has emerged as a prominent generative model in segmentation. This generative model, utilizing weighted variational boundaries, assimilates extensive information during training compared to discriminative models. However, the generative process incrementally condenses the information, occasionally leading to boundary inaccuracies in the resultant segmentations. Baranchuk et al. [19] took the lead in showcasing how DDPM can be integrated into semantic segmentation tasks, extracting superior segmentation performance, especially when working with limited labeled datasets. SegDiff [20] was groundbreaking in its implementation of a two-branch input encoder structure in the diffusion model, which preserved a portion of the gradually compressed semantic data. Rahman et al. [21] capitalized on the model’s stochasticity to glean a plethora of feasible outputs, learning from a segmentation distribution derived from expert panels. Medsegdiff [22] adeptly navigated medical image segmentation challenges by emphasizing specific regions in the diffusion probabilistic model and leveraging dynamic conditional coding with a feature frequency parser. DiffBEV [23] builds upon previous work, harnessing the diffusion model’s denoising capability and introducing a cross-attention module to synergize the autopilot bird’s eye view features with the model’s semantic context. Feng [24] builds TextDiff models by leveraging the ability of diffusion models to capture semantic information and medical text annotations.

In this paper, we apply the diffusion model to the CCTA coronary segmentation task, employing a gated attention mechanism to synchronize the diffusion model data with the segmentation encoder data. The proposed approach is designed to maintain the diffusion model information while ensuring precise coronary segmentation.

### 2.3. U-Net Improvements

U-Net [25], with its encoder–decoder architecture, remains the most preferred framework for semantic segmentation, achieving multi-scale feature fusion by marrying low-level specifics with high-level semantic data via skip connections. Over the years, a slew of refinements has been proposed for U-Net, such as the integration of transformers and attention mechanisms [26]. TransU-Net [4] and CA-Net [27] addresse U-Net’s shortfalls in modeling extensive dependencies and transformers’ inadequacies in low-level nuances by incorporating a self-attention mechanism. Attention U-Net [5] deploys a gated attention mechanism to augment the model’s sensitivity while conserving computational resources. U-NETR [28] conceptualizes the U-Net structure entirely through transformers, transforming 3D medical image segmentation into a sequential prediction task. The Swin transformer [29] pioneers the Shifted windows based on a hierarchical transformer to remedy the semantic disparity between encoder and decoder data overlooked by skip connections. U-Net++ [30] simply piles up multiple skip connections to bridge the semantic gap. MultiResU-Net [31] intensifies the extraction of semantic information by layering multiple residual convolutions on a singular skip connection. CAT-Net [32] evolves the MultiResU-Net’s residual skip connection into a self-attention skip connection, broadening the scope for contextual data aggregation via skip connections. UCTransNet [33] employs a multi-scale information fusion attention mechanism, expanding on previous works.

### 2.4. Large Kernel Convolution

Even though stacking multiple small convolutional kernels can match the receptive field of large convolutional kernels, the ability of large convolutional kernels to extract global and semantic information surpasses that of small convolutional kernels [34]. ConvNeXt [35] delivered top-tier accuracy by introducing a backbone network made up of 7 × 7 convolutional kernels. CNXA [36] advocated for embedding attention mechanisms into ConvNeXt networks using large convolutional kernels. However, employing such large convolutional kernels proves to be complex in training, leading most experiments to utilize dilated convolution or stripe convolution. PDR U-Net [37] uses pure dilated residual convolution to increase the receptive field and realize the automatic segmentation of lower limb bones in X-ray images. RepLKNet [38] attains a span of 31 × 31 with convolutional kernels by paralleling smaller ones. LargeKernel3D [39] authenticates that employing large convolution for 3D vision tasks is both feasible and crucial. LKAU-Net [40] utilizes dilated convolution to assemble a 3D large kernel attention module, addressing variations in brain tumor location, shape, and appearance. SegNext [41] refines the self-attention mechanism using stripe convolution, achieving network performance on par with transformer architectures. SLak [42] leverages sparse matrix decomposition to broaden the convolutional kernel to 51 × 51 through a stripe convolution parallel small convolutional kernel. In this paper, we enhance the network’s capability to extract tubular and contextual information by amalgamating the parallel convolutional kernel trunk with the stripe large convolutional kernel space attention mechanism.

## 3. Our Framework/Coronary Artery Segmentation in CCTA with a Diffusion Model and Spatial Attention

**Figure 1:**
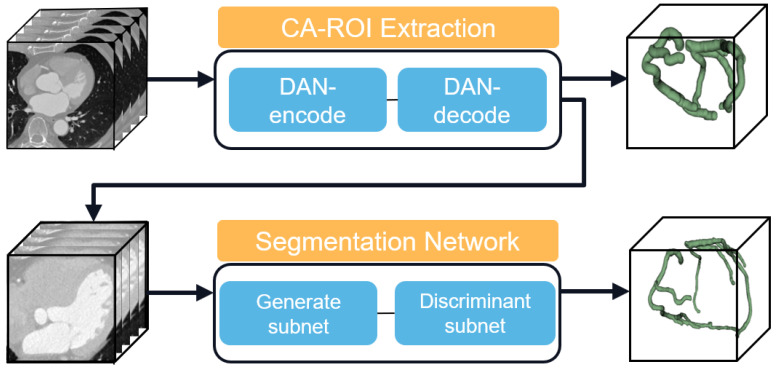
Overall framework process.

Figure 1 illustrates the overall architecture of our proposed automated coronary artery segmentation model. Our method employs a novel hybrid architecture that integrates a generative subnet with a discriminative subnet, facilitating state-of-the-art segmentation performance. This synergistic framework enhances the segmentation by blending probabilistic generation outputs with precise pixel-level classification. The generative subnet adeptly mitigates motion-related artifacts and intrinsic noise characteristics of CTA images. Concurrently, the discriminative subnet, augmented by an innovative attention module, proficiently delineates tubular anatomical structures across varying orientations and scales. This design strategically optimizes the trade-off between model complexity and receptive field coverage, ensuring computational efficiency without compromising on detail capture.

### 3.1. Dilated Attention Network (DAN)

Segmenting coronary arteries (CASs) in CTA images often sets a challenge due to the disparity in volume between the foreground (coronary arteries) and the background. Notably, the cardiac region only comprises about one-eighth of the total volume of a typical CTA image. In order to reduce memory usage and balance foreground–background volumes, we propose to extract the cardiac region through DAN at first. In addition, this is beneficial to reduce disruptions from surrounding pulmonary vessels.

**Figure 2:**
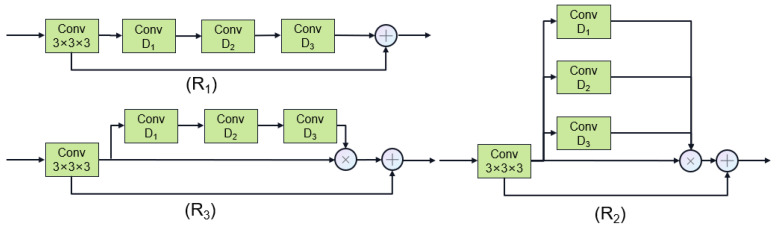
Overall framework process.

The DAN employs the U-Net architecture to extract the coronary region of interest (ROI). Additionally, we introduce two major improvements over traditional U-Net. Firstly, the image was downsampled (512 × 512 × 256 to 128 × 128 × 64) before sending to the DAN, and then the DAN output is upsampled (to 512 × 512 × 256), which ensures the high throughput of our DAN. Secondly, we introduce dilated spatial attention after every convolution layer in U-Net, in order to enlarge the receptive filed with fewer parameters. Three dilation attention structures (R1–R3) with dilation ratios of [1–3] and [1,2,5] are proposed. These ratios were chosen empirically to cover the coronary scale (1–5 mm vessel diameter [16]) while avoiding grid artifacts by conjugate spacing [37]. As shown in Figure 2, the first structure (R1) involves directly stacking dilated convolutions with different ratios. The second (R2) employs parallel dilated convolutions with different ratios, functioning as an attention mechanism. The third (R3) combines stacked dilated convolutions with varying ratios, also serving as an attention mechanism. Figure 2 illustrates these three dilated convolution structures, and their performances will be further discussed in subsequent experiments.

The loss metrics for the network are *Dice* loss and *clDice* loss. The *Dice* loss entails measuring the volumetric overlap between the predicted segmentation and the actual ground truth. The *clDice* loss improves the network’s understanding of the coronary topology. The mathematical representation for the *Dice* loss and *clDice* function is defined as follows:

(1) $$\begin{array}{c} l_{Dice}=1-Dice,\;\;\;\;\;\;\;\;Dice=\frac{2\times \left(V_{L}\cap V_{P}\right)}{\left|V_{L}|+|V_{P}\right|} \end{array}$$

(2) $$\begin{array}{c} l_{clDice}\left(V_{P},V_{L}\right)=2\times \frac{T_{prec}(S_{P},V_{L})\times T_{sens}(S_{L},V_{P})}{T_{prec}\left(S_{P},V_{L}\right)+T_{sens}(S_{L},V_{P})} \end{array}$$

(3) $$\begin{array}{c} T_{prec}\left(S_{P},V_{L}\right)=\frac{\left|S_{P}{\cap V}_{L}\right|}{\left|S_{P}\right|},\;\;T_{sens}(S_{L},V_{P})=\frac{\left|S_{L}{\cap V}_{P}\right|}{\left|S_{L}\right|} \end{array}$$

where $V_{L}$ is the label, $V_{P}$ is the result, $S_{L}$ is the label skeleton, $S_{P}$ is the result skeleton, $T_{prec}$ is the topological accuracy, and $T_{sens}$ is the topological sensitivity.

To extract the ROI, we concatenate DAN’s segmentation results with the original CCTA data, and determine the bounding box based on the segmentation results; then, the bounding box is applied to the original image, which is cropped and upscaled to serve as the input of our next module.

### 3.2. Segmentation Network

#### 3.2.1. Generative Subnet

Following ROI extraction, the primary coronary structures are isolated. However, limitations in contrast agent concentration combined with cardiac and hemodynamic motion introduce significant noise and artifacts.

For this reason, we introduce Denoising Diffusion Probabilistic Models (DDPMs) as the generative subnet, to reduce the noise through learning distributions of CTA images. Another benefit of the diffusion model is that, as a generative model, it can provide better semantic consistency and continuity in semantic segmentation. Generative models can generate high-quality segmentation results with good consistency by learning the global structure and semantic information of images. This helps reduce noise and discontinuities in segmentation results and improves segmentation accuracy and smoothness.

By combining DDPMs with a semantic segmentation model, the generalization of the model to the input image can be promoted. DDPMs can learn the structural and texture features of images and transfer this information in semantic segmentation tasks to improve the model’s perception of details and edges. This helps improve the performance of segmentation models on complex scenes and different datasets.

Our generative subnet draws its foundation from the Deep Diffusion Probabilistic Models (DDPMs). This diffusion segmentation component is bifurcated into two phases: the forward diffusion and the backward diffusion. The former involves iteratively perturbing the input with Gaussian noise across multiple steps. Conversely, the latter phase involves a methodical reverse diffusion, aiming to restore the initial input data incrementally. Both these phases can be conceptualized as parameterized Markov chains [43,44,45,46].

In the forward process, we use the features generated by the decoder as the condition for the diffusion model. Gaussian noise is gradually added to the original image $x_{0}$ through a series of T steps. The forward process can be expressed as follows:

(4) $$\begin{array}{c} q\left(x_{t}|x_{t-1}\right)=𝒩\left(x_{t};\sqrt{1-\beta_{t}}x_{t-1},\beta_{t}I\right) \end{array}$$

where *β_t* is a pre-defined variance sequence. We can directly calculate $x_{t}$: from the given $x_{0}$:

(5) $$\begin{array}{c} q\left(x_{t}|x_{0}\right)=𝒩\left(x_{t};\sqrt{\bar{\alpha_{t}}}x_{t0},\left(1-\bar{\alpha_{t}}\right)I\right) \end{array}$$

where $\alpha_{t}≔1-\beta_{t}$ and $\bar{\alpha_{t}}≔\prod_{s=1}^{t}\alpha_{s}$

In the backward process, a neural network is trained to recover the original data by reversing the noise process, which can be expressed as follows:

(6) $$\begin{array}{c} p_{\theta }\left(x_{0:T-1}|x_{T}\right)=\prod_{t=1}^{T}p_{\theta }\left(x_{t-1}|x_{t}\right) \end{array}$$

Here, θ is the parameter of the reverse process. In the backward process, starting from Gaussian noise, $p_{\theta }\left(x_{T}\right)=N\left(x_{T};0,I_{n\times n}\right)$, where I represents the original image and transforms the latent variable distribution $p_{\theta }\left(x_{T}\right)$ into the data distribution $p_{\theta }\left(x_{0}\right)$. To maintain symmetry with the forward process, the backward process gradually recovers the noisy image to obtain a final clear segmentation.

**Figure 3:**
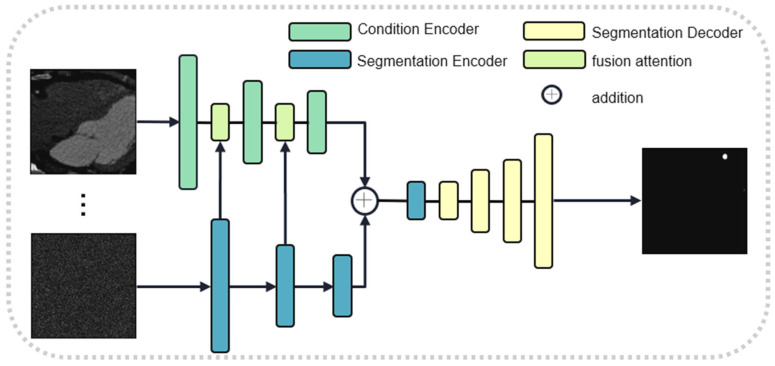
Generative subnet.

To prevent the progressive compression loss of information during the stochastic noise addition process of the diffusion model, we introduce a dual-encoder structure. One encoder implements the diffusion model, while the other encoder utilizes a segmentation network to extract features. These two types of features are adaptively fused together using a gate attention mechanism. Since the two features do not have significant shape differences, we have removed the scaling stage of the original gate attention mechanism. The structures of the diffusion network and the gate attention mechanism are illustrated in Figure 2 and Figure 3, respectively. We use U-Net as the learning network and estimate the step size function ϵ with the prior condition of the original image, which can be expressed as follows:

(7) $$\epsilon_{\theta }\left(x_{t},I,t\right)=D\left(\left(E_{t}^{I}+E_{t}^{x},t\right),t\right),$$

Here, $E_{t}^{I}$, provided by the DAN, is a 3D feature. It is used as a conditional input to constrain the 2D diffusion model for mask generation. $E_{t}^{x}$ is the 2D feature embedding of the segmentation mapping at the current step. The two features are added together and sent to the U-Net decoder for reconstruction.

Notably, although the generative subnet generates 2D probabilistic masks slice-by-slice, these masks are integrated into the 3D segmentation pipeline through a cross-slice contextual fusion mechanism in the discriminative subnet. Instead of processing each slice independently, the 2D masks are concatenated with 3D features from the encoder at each hierarchical level, allowing the model to fuse slice-wise details with volumetric spatial dependencies. This design provides volumetric context to ensure that the 2D masks contribute to coherent 3D segmentation by leveraging inter-slice feature propagation in the subsequent attention modules (detailed in Section 3.2.2).

#### 3.2.2. Discriminant Subnet

**Figure 4:**
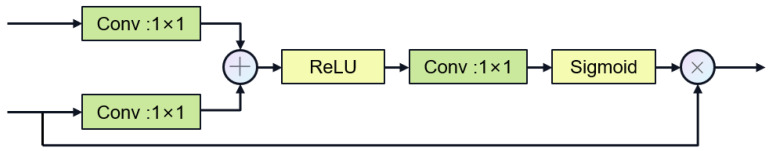
Gate attention.

**Figure 5:**
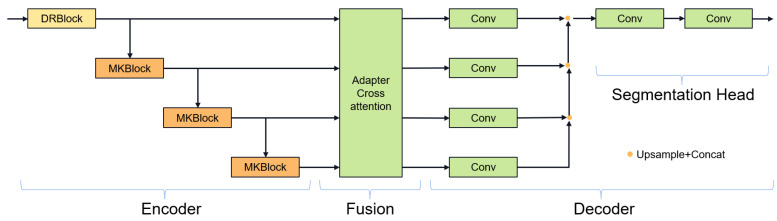
Fine segmentation overall structure.

In the latest research, e.g., MultiResU-Net [31] and UCTransNet [33], it has been demonstrated that the encoder–decoder architecture of U-Net will lead to semantic gaps between the encoder and the decoder. Inspired by 2D FFnet [47], we propose a new segmentation network structure aiming to alleviate the above problems. Figure 4 showcases the gate attention mechanism, which plays a crucial role in guiding feature processing. Our network design is shown in Figure 5, which decomposes the encoder–decoder architecture into encoder, feature fusion, and decoder modules. First, the encoder module is responsible for extracting high-level feature representations of the input image. Then, the feature fusion module fuses the multi-layer features of the encoder to enhance the transfer and integration of semantic information. Finally, the decoder module generates the final segmentation results through feature decoding and segmentation heads.

**Figure 6:**
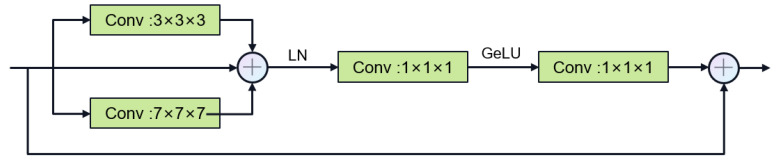
Multi-Kernel Block.

Encoder process: We designed to use two types of convolution blocks. Firstly, the first layer uses the DenseRes block, inspired by DRU-Net [47]. An additional connection is added between the output of the first convolutional BN operation and the output of the last convolutional BN operation, and the feature maps are aggregated by a summation operation, which combines the advantages of ResNet and DenseNet. This convolution block does not add extra parameters and can ensure that the output of the first convolutional layer is not affected even if the second convolutional layer encounters gradient vanishing. In the decoder, we constructed the Multi-Kernel block as shown in Figure 6, which uses 3 × 3 × 3 and 7 × 7 × 7 convolution kernels in parallel. Using large convolution kernels can ensure a sufficiently large receptive field for modeling global vessel continuity and enhance contextual awareness and is inspired by ConvNext [35], which can achieve excellent local feature extraction capabilities and long-distance dependency capabilities not inferior to transformers. The parallel use of small convolution kernels reduces the difficulty of training and supplements local information extraction. We use two types of residual connections: Post norm and Pre norm [48]. Post norm normalizes the output after the residual operation, which has a stronger effect on parameter regularization and can improve the robustness of the model. Pre norm, on the other hand, does not require regularization for some parameters because they are added directly to the output, which can prevent gradient explosion or vanishing.

Feature fusion stage: We use multi-stage gate attention mechanism for feature fusion and add the large convolutional kernel stripe spatial attention mechanism and channel attention mechanism in the deepest layer of the network. For channel attention, we use ECAnet [49], which extracts channel information from feature maps by using global average pooling. Information interaction between channels is achieved by introducing shared parameter convolution without reducing the dimensionality. The calculation of channel attention can be summarized as follows:

(8) $$\begin{array}{c} CA\left(F\right)=\sigma \left({AvgPool\left(F\right)}^{T}Conv\left(AvgPool\left(F\right)\right)\right) \end{array}$$

**Figure 7:**
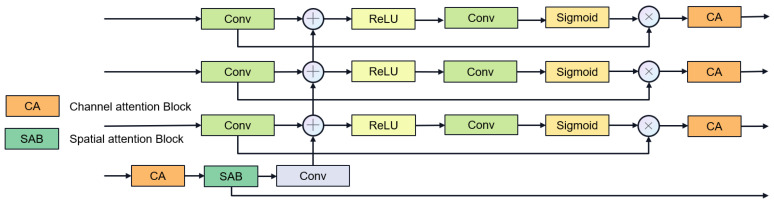
Multi-stage gated attention mechanism.

**Figure 8:**
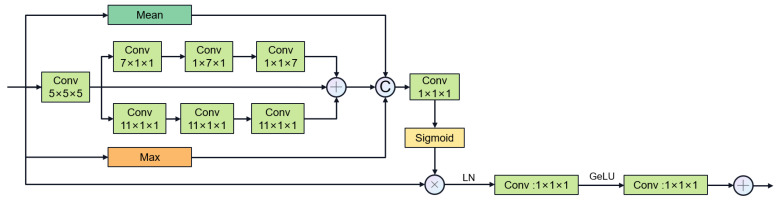
Stripe large convolutional kernel spatial attention mechanism (“C” stands for Concatenation.).

The multi-stage gated attention mechanism as shown in Figure 7 further enhances the feature fusion capability by integrating spatial attention and channel attention. Furthermore, inspired by segNext [41], we constructed the large convolutional kernel stripe spatial attention mechanism, as shown in Figure 8. DSCNet [17] and AVDNet [50] have proved that stripe convolution can retain more spatial information, reduce sensitivity to structural changes, and improve the network’s perception and recognition accuracy of tubular structures. Specifically, the mechanism employs asymmetric 3D convolution kernels (e.g., 1 × 7 × 1, 7 × 1 × 1) to capture longitudinal features along the vessel axis across slices, explicitly modeling inter-slice continuity for coronary arteries. Compared with dynamic convolution kernels, stripe convolution does not add additional calculations during the training process and can maintain high efficiency. We first use average pooling and max pooling to preserve key channel information, then use asymmetric convolution to collect contextual spatial information, and finally use 1 × 1 × 1 convolution kernel to aggregate channel information. The calculation of spatial attention can be summarized as follows:

(9) $$\begin{array}{c} LKA\left(F\right)=\sigma \left(Conv(concat(Mean\left(F\right),Max\left(F\right),DWconv(F))\right) \end{array}$$

Decoder stage: U-Net performs one feature fusion followed by one decoding, where all features are first fused before decoding each level of fused features. Because feature fusion and the decoder are separated, using U-Net’s single output structure can cause deep-level features to not participate in the backpropagation process, so a deep supervision method is needed, where the decoded features at each level are merged and connected and finally output through a segmentation head.

Loss function: Though we alleviate the foreground–background imbalance problem through ROI extraction in Section 3.1, this remains a challenge for successive modules. To solve this problem, we use Dice loss and Focal loss [51] in SAN. The Focal loss reduces the weight of easily classified samples by introducing a balance factor and a focus factor, thereby making the model pay more attention to difficult samples. This can effectively reduce the contribution of easy samples to training loss and increase attention to difficult samples. The formula of the Focal loss is defined as follows:

(10) $$\begin{array}{c} L_{Focal}\left(p_{t}\right)=-{\left(1-p_{t}\right)}^{\gamma }\log\left(p_{t}\right) \end{array}$$

Among the variables, $p_{t}$ represents the degree of similarity to the label and γ is an adjustable factor.

## 4. Experimental Result and Analysis

### 4.1. Datasets and Preprocessing

The ImageCAS dataset [16] is a publicly available large-scale coronary artery segmentation dataset consisting of 1000 cardiac computed tomography angiography (CCTA) images. The dataset was divided into training and testing sets through random selection. The original images in the dataset have a resolution of 512 × 512 × *N*, where *N* ranges from 206 to 275. The in-plane resolution is 0.29~0.43 mm^2^, and the slice thickness ranges from 0.25~0.45 mm. The dataset includes 414 female and 585 male patients, with average ages of 59.98 and 57.68 years, respectively.

The ImageLaPP dataset is an internal dataset consisting of 80 CCTA images. We divided the dataset into a training set of 60 images, a validation set of 10 images, and a test set of 10 images. The left and right coronary arteries in each image were labelled independently by two radiologists and the results were cross-validated. In case of disagreement, a third radiologist provided annotations, and the final annotations were based on a consensus. The original images in the dataset have a resolution of 512 × 512 × N, where N ranges from 200 to 380.

To improve the visibility of coronary artery tissue and to remove the influence of lung noise information, we constrained the CT values to the range [−90, 410] HU. We then merged the constrained images and the original images as input channels. In addition, we applied a truncation normalization to all images.

### 4.2. Parameter Setting

All experiments were conducted using the PyTorch 1.12.1 framework on an Ubuntu 16.04.4 LTS system with an Nvidia RTX 3090 Ti GPU. The ImageCAS dataset was randomly divided into training, testing, and validation sets in a 7:1:2 ratio. During coronary artery ROI extraction, we employed the *Dice* loss and *clDice* loss functions for training, utilizing the AdamW optimizer with a fixed learning rate of 0.001 for 50 training epochs.

The generative subnet was configured with 1000 diffusion steps, a learning rate of $5\times 10^{-5}$, a batch size of 2, and 50,000 total iterations. The discriminative subnet (SAN) used a batch size of 1 for 50 epochs, initialized with a learning rate of 0.001 and a minimum learning rate of $1\times 10^{-6}$, employing a cosine annealing learning rate decay strategy. This memory-constrained setup is necessitated by memory constraints imposed by 3D volume data, as smaller batches are required to fit large medical volumes into GPU memory [Roth et al., MedIA 2020] [52]. To address potential gradient noise from small batches, gradient accumulation and cosine annealing learning rates were employed to stabilize training and ensure convergence. Throughout the experiments, consistent system environments, hardware models, and parameter settings were maintained to ensure fair comparisons among all models. All evaluation metrics reported in the paper represent average values.

### 4.3. Evaluation Indicators

This study introduced the Dice similarity coefficient as a metric for objectively comparing experimental models. The Dice coefficient quantifies the level of overlap between segmentation results and ground truth annotations, with a higher value indicating a greater degree of similarity between the two. The formula for calculating the Dice similarity coefficient (DSC) is as follows:

(11) $$\begin{array}{c} DSC=\frac{2\times \mathrm{card}\left(A\cap B\right)}{\mathrm{card}\left(A\right)+\mathrm{card}\left(B\right)}=\frac{2TP}{FP+FN+2TP} \end{array}$$

TP and FP represent a true positive and false positive, respectively, indicating the number of voxels correctly segmented as vessels and the number of background voxels incorrectly segmented as vessels by the model. Additionally, FN represents the false negative variable, indicating the number of vessel voxels incorrectly labeled as background voxels.

Given the significance of boundary accuracy in vessel segmentation and the limitations of the Dice coefficient in measuring spatial position, topological structure, and other segmentation result details, this study introduced the Hausdorff distance (HD) metric. The Hausdorff distance is a metric used to calculate the maximum distance between any point in the segmentation result and the corresponding point in the ground truth annotation. A smaller HD value indicates a closer proximity between the segmentation result and the ground truth annotation, resulting in higher segmentation accuracy. The formula to compute the HD metric is as follows:

(12) $$\begin{array}{c} HD=max\left(\underset{a\in A}{max}\{\underset{b\in B}{min}||a-b||\},\underset{b\in B}{max}\{\underset{a\in A}{min}||b-a||\}\right) \end{array}$$

Here, A and B denote the boundaries of the segmented object and the mask, respectively.

Due to the maximum HD’s sensitivity to noise and outliers, this study introduced the average Hausdorff distance (AHD) metric, which calculates the average Hausdorff distance across multiple samples, effectively eliminating the influence of outliers. The AHD metric serves as a measure of the overall performance of segmentation algorithms, where smaller values correspond to superior overall segmentation algorithm performance.

Among these metrics, the Dice coefficient mainly evaluates the performance of the segmentation method. In this study, we primarily rank the results based on the Dice coefficient, followed by AHD and HD.

### 4.4. Comparative Experiment

In this subsection, we perform a comparative evaluation of our proposed method against ten state-of-the-art approaches. These approaches are categorized into three groups based on their architectural characteristics and application domains. The first group comprises general-purpose convolutional deep learning networks, namely U-Net3D [24,53], FFnet [22], and V-Net [54], which have a demonstrated effectiveness in various segmentation tasks. The second group consists of segmentation networks that leverage transformer or similar structures, including TransU-Net [4], U-NETR [28], and 3D UX-Net [55]. These models have been specifically designed to exploit the strengths of attention mechanisms for improved segmentation performance. The third group encompasses deep learning networks explicitly tailored for vessel segmentation, which include Attention-FCN [7], DSCNet [17], DenseVoxNet [56], and the ImageCAS baseline. These models adopt specialized architectural designs to address the unique challenges associated with vessel segmentation tasks. To ensure a fair and unbiased comparison, we employ consistent data preprocessing procedures across all experiments. Moreover, we retrain all compared models using the same learning strategy to ensure optimal performance.

#### 4.4.1. Quantitative Results

Table 1 presents a comprehensive quantitative evaluation of our proposed method and ten comparative approaches on the ImageCAS dataset. The experimental results demonstrate the superior performance of our 3D network in coronary artery segmentation (CAS). Specifically, our method achieves an average Dice similarity coefficient (DSC) of 83.48%, a Hausdorff distance (HD) of 19.43 mm, and an average Hausdorff distance (AHD) of 0.6007 mm. These metrics outperform all other methods, highlighting the effectiveness and accuracy of our model. Compared to the classical U-Net3D model, our method exhibits substantial improvements. The DSC score is enhanced by approximately 6.57, while the mean values of HD and AHD are reduced by about 14.8233 mm and 0.0697 mm, respectively. These enhancements further underscore the validity and precision of our model. Among the other two groups of comparative methods, namely transformer-based methods and design-specific methods, U-NETR and ImageCAS demonstrate the best performance, respectively. Notably, our method surpasses both U-NETR and ImageCAS in terms of DSC scores by 5.6% and 0.72%, respectively.

**Figure 9:**
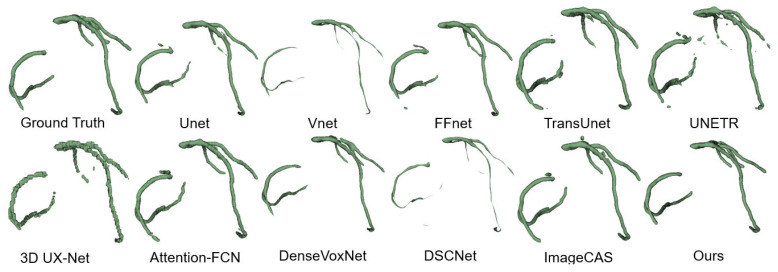
ImageCAS dataset segmentation results.

Results are reported as mean values due to the limited sample size of clinical datasets. While statistical tests (e.g., standard deviation, *p*-values) are omitted, the observed performance gains (e.g., +0.74% DSC over ImageCAS baseline) reflect consistent improvements across multiple trials, as validated by qualitative visualizations (Figure 9) and ablation studies.

Overall, our method exhibits superior reliability in terms of DSC, HD, and AHD. It outperforms other models in terms of segmentation accuracy and boundary precision. In particular, DSCNet outperforms our method in HD and AHD, but is worse than our method in DSC. This is primarily due to the utilization of dynamic convolution in the DSCNet, which closely fits the thin and curved tubular structure of coronary arteries. In contrast, our method employs strip convolution to approximate tubular information, prioritizing multi-view global morphological information over boundary details. These findings collectively demonstrate the effectiveness and accuracy of our proposed method in coronary artery segmentation, highlighting its ability to capture essential morphological information while achieving competitive boundary accuracy.

#### 4.4.2. Qualitative Results

We employed the 3D Slicer toolbox to visualize and compare the qualitative results of the CAS segmentation. Figure 9 illustrates the visualizations of the coronary artery segmentation by various methods. While most methods demonstrate the accurate segmentation of the thicker vessels in the coronary arteries, challenges arise when dealing with branch structures. Convolutional deep learning networks tend to lose branch structures and suffer from overfitting, while transformer-based networks often encounter fractures in the segmentation results. In contrast, our proposed method consistently achieves a more accurate 3D structure that closely resembles the ground truth across most data samples.

Through a comprehensive analysis of both quantitative and qualitative results, our proposed approach exhibits significant and consistent performance improvements. These advancements can be attributed to the synergistic effect achieved by seamlessly integrating our proposed spatial attention and diffusion models. The integration effectively addresses the challenges posed by the substantial noise and complex semantics present in CAS datasets. Our approach demonstrates remarkable effectiveness in overcoming these challenges and achieving high-quality segmentation results.

### 4.5. Generalization Experiment

In the field of medical image processing, the generalization ability of a deep learning model plays a crucial role. A deep learning model with good generalization ability can effectively learn common features and patterns from limited training data, enabling accurate prediction and analysis on previously unseen data. This ability is particularly important in medical applications where data diversity and limited sample sizes are common. A model that exhibits strong generalization can adapt to different data distributions and variations, enhancing the reliability and practicality of the model.

**Figure 10:**
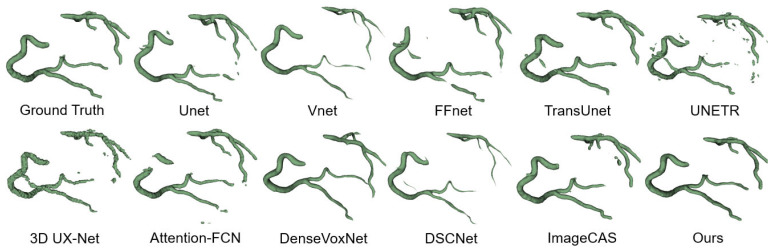
Segmentation results of ImageLaPP dataset.

To evaluate the generalization ability of our proposed network, we conducted experiments by testing the model trained on the ImageCAS dataset on the ImageLaPP dataset. The results, as presented in Table 2 and Figure 10, demonstrate the optimal segmentation performance achieved by our model on the ImageLaPP dataset. These findings indicate that our proposed model possesses a strong generalization ability for coronary artery segmentation (CAS). By successfully performing well on unseen data from a different dataset, our model showcases its capability to generalize beyond the training data and perform effectively in diverse clinical scenarios.

### 4.6. Ablation Studies

#### 4.6.1. DAN

**Figure 11:**
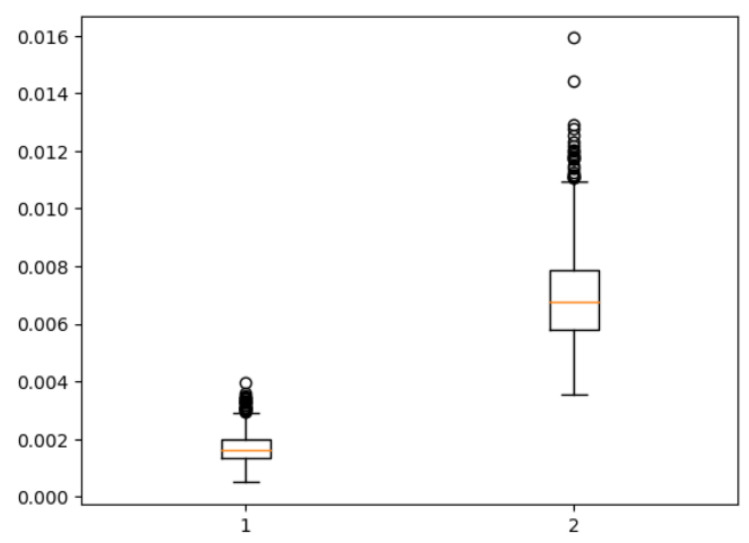
Foreground–background ratio before and after ROI extraction.

**Figure 12:**
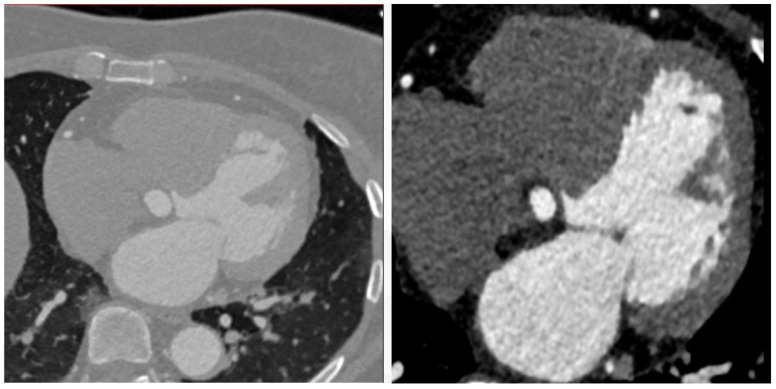
Comparison of pictures before and after ROI extraction.

Figure 11 presents a boxplot depicting the ratio of coronal voxels to background voxels before and after the extraction of the region of interest (ROI). It is evident that the application of clipping leads to a significant increase in the foreground-to-background ratio, approximately quadrupling its value. This demonstrates the effectiveness of ROI extraction in addressing the severe foreground–background imbalance issue. Figure 12 provides a qualitative assessment of the images before and after ROI extraction. The visual comparison validates the substantial impact of ROI extraction in addressing the foreground–background imbalance problem. The images clearly demonstrate that the ROI extraction operation effectively mitigates the imbalance, resulting in a more accurate representation of the regions of interest.

Table 3 presents the results obtained from employing three different structures of expansion space attention in the DAN network, using various expansion rate parameter settings. The findings indicate that the structure labeled as R3 proves advantageous for efficient ROI extraction through deep learning. This can be attributed to the fact that dilated convolutions excel in extracting spatial shapes but have limited ability in capturing detailed textures. By utilizing dilated convolutions, the proposed structure effectively preserves the spatial information while avoiding the suppression of previously learned detailed features. Consequently, the R3 structure facilitates the rapid extraction of ROIs and enhances the overall performance of the deep learning framework.

#### 4.6.2. Diffusion Model

**Figure 13:**
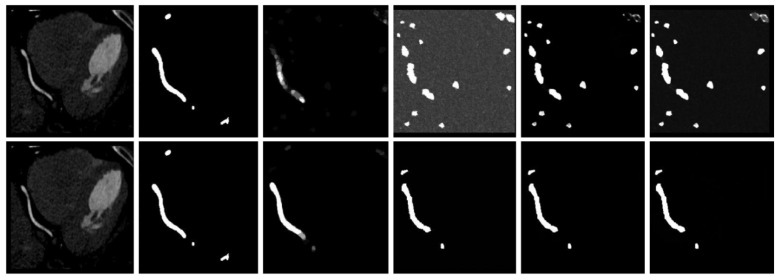
Comparison of the results in the generation stage. The upper layer is the original image, real label, average result, probability generation result 1, probability generation result 2, and probability generation result 3 when the diffusion step is 50, and the lower layer is the original image when the diffusion step is 1000, real label, average result, probability generation result 1, probability generation result 2, and probability generation result 3.

In Table 4, we examine the influence of the generative subnet on the overall framework. Our generative subnet uses the stochastic diffusion learning process of DDPM, and thus has a significant influence on the model. The choice of T = 1000 aligns with DDPM’s theoretical foundation [18], ensuring the noise schedule fully corrupts the input while maintaining stable reverse-process learning. The 1.1% DSC drop at T = 50 confirms that insufficient steps degrade diffusion quality, as predicted by [22]. Figure 13 shows a significant discrepancy between the generated probabilistic masks and the true labels when the diffusion step is only 50. We note that this discrepancy may be due to the suboptimal selection of the diffusion step for the given dataset, resulting in the model’s denoising capability falling short of its optimal potential. A smaller diffusion step means that the model takes fewer diffusion steps in a shorter time. This may cause the model to be unable to fully utilize all the information in the input image during the denoising process, resulting in the loss or incomplete propagation of information.

#### 4.6.3. SAN

We conducted ablation experiments on the discriminative subnet using the ImageCAS dataset, incorporating multi-scale convolutional blocks, a multi-order gated feature fusion attention mechanism, and a large convolutional kernel strip space attention mechanism. The results in Table 5 demonstrate the positive impact of the network enhancements on the coronary segmentation task. Furthermore, we evaluated the effect of different loss functions through ablation experiments, and Table 6 reveals that both the skeleton loss function and the Focal loss function yield beneficial effects on the coronary segmentation task, with the Focal loss function exhibiting superior performance.

### 4.7. Clinical Translation of Metrics

The quantitative performance of SADiff demonstrates direct clinical utility in three key dimensions:

Vessel Diameter Estimation: The HD of 0.60 mm corresponds to subvoxel-level accuracy (ImageCAS voxel size: 0.29–0.43 mm). Prior studies indicate that boundary errors below 1.0 mm are sufficient for reliable stenosis detection (>50% lumen narrowing) in CCTA [2]. Our results fall well within this tolerance, suggesting clinically acceptable diameter measurement precision.

FFR Simulation Feasibility: Segmentation continuity (evident in Figure 9) minimizes artifacts in computational fluid dynamics (CFD) pipelines. The literature demonstrates that HD < 1.0 mm ensures FFR prediction errors within 0.05—a threshold for clinical relevance [2,50]. Our HD of 0.60 mm aligns with these requirements.

Radiologist Workload: The 6.57% DSC improvement over U-Net3D suggests enhanced segmentation consistency, as evidenced by fewer fragmented vessels in Figure 9. This improvement likely translates to reduced manual corrections, though direct time savings require further clinical validation.

These advances have made the SADiff a viable tool for the accurate and efficient assessment of coronary arteries in the clinical setting.

## 5. Discussion and Conclusions

In this study, we proposed a novel deep learning network specifically designed for coronary segmentation in CTA images. Our approach addresses the challenges posed by noise factors, such as clinical variability and motion artifacts, by integrating a generative diffusion model with a convolutional segmentation model. To efficiently extract global information and the region of interest (ROI), we employed a dilated convolutional attention mechanism within the dilated attention network (DAN). Meanwhile, the diffusion model was utilized to capture local information within the ROI, effectively suppressing the detrimental effects of noise, including motion artifacts. By incorporating both global and local information, the strip-convolutional attentional mechanism within the strip attention network (SAN) accommodates the tubular structure characteristic of the coronary arteries. Our proposed method was thoroughly evaluated on the ImageCAS dataset, demonstrating its effectiveness and general applicability. Furthermore, we assessed the generalization performance of our model on the ImageLaPP dataset, highlighting its robustness across different data sources. The results of our study establish our method as an effective solution for coronary segmentation, significantly advancing the field of deep learning in this domain.

Discussion of the limitations: Our model still has some limitations. While the masks generated by the diffusion model can partially fill in the difficult-to-segment structures, excessively random mask generation can introduce additional noise into the discriminative model, leading to a decrease in segmentation performance. Furthermore, the generation efficiency of the diffusion model is low and consumes a significant amount of GPU resources. In addition, this study did not consider the challenges of the difficulty of annotating CCTA data and the scarcity of publicly available large-scale datasets. Future advancements in GPU computational capacity and the increasing availability of large-scale medical datasets are expected to mitigate these limitations.

Discussion of our future work: In the future, we plan to use semi-supervised methods and hybrid attention mechanisms to improve the segmentation capability of the network, and we may also explore memory-efficient 3D DDPM architectures [35] to directly process 3D volumes and enhance inter-slice consistency. In addition, we plan to introduce generic segmentation models, such as SAM [57], to reduce the training cost for extracting coronary ROIs.

## Figures and Tables

**Table 1:** ImageCAS dataset performance comparison. (“↑” means that the higher the better, and the higher the value, the better the segmentation effect on this indicator. “↓” means that the lower the better, and the smaller the value, the higher the segmentation accuracy.)

Method	Input Size	DSC Score (%) ↑	HD (mm) ↓	AHD (mm) ↓
3D U-Net	256 × 256 × 128	76.91	34.2554	0.6704
Vnet	256 × 256 × 128	75.31	27.5506	0.6798
FFnet	256 × 256 × 128	76.87	20.5529	0.6231
TransU-Net	256 × 256 × 128	76.12	28.5101	0.9316
UNTER	256 × 256 × 128	77.88	41.0667	0.9348
3D UX-Net	256 × 256 × 128	76.16	30.1137	0.9835
Attention-FCN	256 × 256 × 128	76.05	26.2148	0.7957
DenseVoxelNet	256 × 256 × 128	77.72	27.2357	0.9963
DSCNet	256 × 256 × 128	79.24	15.9039	0.5069
ImageCAS	128 × 128 × 64, 163,323,643	82.74	27.1289	0.8381
SADiff	256 × 256 × 128 256 × 256	83.48	19.4321	0.6007

**Table 2:** ImageLaPP dataset performance comparison.

Method	Input Size	DSC Score (%) ↑	HD (mm) ↓	AHD (mm) ↓
3D U-Net	256 × 256 × 128	76.27	38.3548	0.7784
Vnet	256 × 256 × 128	76.01	28.5184	0.7412
FFnet	256 × 256 × 128	76.18	23.9421	0.7764
TransU-Net	256 × 256 × 128	75.33	29.5405	0.9982
UNTER	256 × 256 × 128	77.86	43.7563	1.0215
3D UX-Net	256 × 256 × 128	77.54	32.5816	0.9754
Attention-FCN	256 × 256 × 128	75.17	31.1182	0.9457
DenseVoxNet	256 × 256 × 128	76.85	27.2357	0.8981
DSCNet	256 × 256 × 128	78.71	18.2419	0.6118
ImageCAS	128 × 128 × 64, 163,323,643	78.87	30.5516	0.8325
SADiff	256 × 256 × 128 256 × 256	79.42	22.6284	0.7501

**Table 3:** Comparison three different attention structure networks.

R_1_	R_2_	R_3_	DSC Score (%) ↑	HD (mm) ↓	AHD (mm) ↓
1,2,3			77.96	31.1721	0.8792
1,2,5			77.42	31.0834	0.9111
	1,2,3		78.4	28.3864	0.8013
	1,2,5		78.26	29.2643	0.8028
		1,2,3	78.99	27.0866	0.7425
		1,2,5	79.01	26.2473	0.8329

**Table 4:** Comparison of minimal (T = 50) and standard (T = 1000) steps.

Step	DSC Score (%)↑	HD (mm)↓	AHD (mm)↓
50	80.33	45.9501	0.9265
1000	81.43	30.1126	0.8331

**Table 5:** Fine segmentation stage ablation experiments.

	FFnet	Multi-Kernel Block	Multi-Stage Gated Attention	Strip Spatial Attention	DSC Score (%)↑	HD (mm) ↓	AHD (mm)↓
1	√				81.87	26.2366	0.8152
2	√	√			82.01	21.5543	0.7529
3	√		√		81.97	24.9755	0.7966
4	√			√	82.14	21.9588	0.7416
5	√	√	√		82.18	23.1745	0.7015
6	√	√		√	82.24	21.3149	0.6953
7	√		√	√	82.89	20.4752	0.6681
8	√	√	√	√	83.48	19.4321	0.6007

**Table 6:** Loss function ablation experiment.

Loss.	DSC Score (%)↑	HD (mm)↓	AHD (mm)↓
dice loss	83.17	22.8244	0.9521
cldice + dice loss	83.46	21.5993	0.7016
focal + dice loss	83.48	19.4321	0.6007

## Data Availability

Data available on request from the authors.
